# Site‐specific treatment outcome in smokers following non‐surgical and surgical periodontal therapy

**DOI:** 10.1111/jcpe.12462

**Published:** 2015-10-28

**Authors:** Dagmar F. Bunæs, Stein Atle Lie, Morten Enersen, Anne Nordrehaug Aastrøm, Kamal Mustafa, Knut N. Leknes

**Affiliations:** ^1^ Faculty of Medicine and Dentistry Department of Clinical Dentistry University of Bergen Bergen Norway; ^2^ Faculty of Dentistry Institute of Oral Biology University of Oslo Oslo Norway

**Keywords:** chronic periodontitis, cluster analysis, microbiology, periodontal therapy, smoking

## Abstract

**Aim:**

To evaluate the effect of smoking at patient, tooth, and site level following non‐surgical and surgical periodontal therapy.

**Material and Methods:**

Eighty chronic periodontitis patients, 40 smokers and 40 non‐smokers, were recruited to this single‐arm clinical trial. Smoking status was validated by measuring serum cotinine levels. Periodontal examinations were performed at baseline (T0) and 3 months following non‐surgical and surgical periodontal therapy (T1). At T0 and T1, subgingival plaque samples were collected from the deepest periodontal pocket in each patient and analysed using checkerboard DNA–DNA hybridization. Probing depth (PD) ≥ 5 mm with bleeding on probing (BoP) was defined as the primary outcome. Unadjusted and adjusted logistic regression analyses, corrected for clustered observations within patients and teeth, were conducted comparing smokers with non‐smokers.

**Results:**

Clinical parameters significantly improved in both groups (*p *<* *0.001). An association was revealed between smoking and PD ≥ 5 mm with BoP (OR= 1.90, CI: 1.14, 3.15, *p *=* *0.013), especially for plaque‐positive sites (OR= 4.14, CI: 2.16, 7.96, *p *<* *0.001). A significant reduction of red complex microbiota was observed for non‐smokers only (*p *=* *0.010).

**Conclusion:**

Smokers respond less favourably to non‐surgical and surgical periodontal therapy compared with non‐smokers, in particular at plaque‐positive sites.

Cigarette smoking appears a considerable behavioural risk factor for periodontal diseases (Albandar 2002). Depending on the definition of the disease and exposure to smoking, a smoker has 3–25 times higher risk of developing chronic periodontitis compared with non‐smokers (Bergström 2003, Hyman & Reid 2003). Nearly half of the cases diagnosed with chronic periodontitis are smokers (Tomar & Asma 2000, Hyman & Reid 2003, Do et al. 2008). In perspective, there is a globally increasing prevalence of cigarette smokers (Samet & Wipfli 2010, Ng et al. 2014).

Chronic periodontitis patients generally respond favourably to conventional periodontal treatment. However, several studies indicate that smokers respond less favourably both to non‐surgical (Preber & Bergström 1986, Apatzidou et al. 2005, Wan et al. 2009) and surgical approaches (Trombelli et al. 1997, 2003, Scabbia et al. 2001). In spite of this, smokers are in general treated following similar protocols as for non‐smokers.

Cigarette smoking is considered an extrinsic modifying factor in the pathogenesis of periodontal diseases interacting with the host cells and affecting the inflammatory response to microbial challenge (Palmer et al. 2005). Plausible pathognomonic mechanisms include impaired neutrophil function, decreased lymphocyte proliferation and IgG production, altered release of cytokines (Al‐Shammari et al. 2001, Orbak et al. 2003, Persson et al. 2003, Apatzidou et al. 2005), reduced revascularization, and decreased fibroblast proliferation, attachment and collagen synthesis (Gamal & Bayomy 2002, Mavropoulos et al. 2007, Semlali et al. 2011). Smokers also harbour increased levels of putative periodontal pathogens compared with non‐smokers (Haffajee & Socransky 2001b, Van Winkelhoff et al. 2001, Guglielmetti et al. 2014, Joshi et al. 2014). Interestingly, the periodontal microbiota in smokers may return to normal within 6–12 months following smoking cessation (Fullmer et al. 2009).

The compromising effect of cigarette smoking on periodontal therapy appears dose‐dependent (Kaldahl et al. 1996, Rieder et al. 2004). Thus, an objective estimation of smoking status emerges as an important prerequisite to identify and assess any harmful effects of smoking on the periodontium at a patient, tooth, and site level (Scott et al. 2001, Kotsakis et al. 2015). The use of self‐reported smoking data is prone to bias in individuals who often are unwilling to disclose their smoking status. Therefore, self‐reported smoking status needs to be objectively validated.

Besides the systemic effect, smoking may also exert local effects. Palatal sites and molar teeth seem to be more susceptible to advanced attachment loss throughout disease progression (Haffajee & Socransky 2001a). To predict the outcome of periodontal therapy in smokers, the effect of smoking needs to be explored at patient, tooth, and site level. As variations in periodontal treatment outcomes to a great extent are explained by factors acting at a site level (D'Aiuto et al. 2005, Kim et al. 2007), the application of statistical models analysing sites, taking the clustering of data over teeth and patients into account, appears appealing. Focusing on the effects at particular sites may provide a more accurate explanation of the natural hierarchical structure of the treatment responses following periodontal therapy.

There seem to be no prospective studies evaluating the effect of cigarette smoking on the outcomes of non‐surgical and surgical periodontal treatment at site level in chronic periodontitis patients, corrected for clustered observations. The overall purpose of this study was to compare the initial periodontal treatment outcome in smokers and non‐smokers. More specifically, the aims were to evaluate the effect of smoking at patient, tooth, and site level following non‐surgical and surgical periodontal therapy and to compare differences in the composition of the subgingival microflora during treatment at the patient level.

## Material and Methods

### Pre‐study protocols and tests

The study protocol and informed consent approved by the Institutional Medical Research Ethics Committee (2011/151‐6), University of Bergen, Norway followed the Helsinki Declaration of 1975, version 2008. Prior to inclusion, all patients read and signed a written consent form.

### Intra‐examiner calibration

A calibration exercise was performed to obtain intra‐examiner reproducibility for the clinical outcome variable probing depth (PD) and clinical attachment level (CAL). In a sample of 10 subjects, PD and CAL were measured twice, 1 day apart, at six sites per tooth and the intraclass correlation coefficients (ICC) were calculated separately for each site. The ICC ranged between 0.92 and 0.96 for PD and between 0.93 and 0.96 for CAL.

### Sample size

The sample size estimation was based on change in PD. A difference of 0.5 mm from T0 to T1 was considered clinically relevant. Standard deviation of the differences between repeated PD measurements from the intra‐calibration exercise was 0.5 mm. A power analysis based on 40 subjects per group and with the level of significance (*α*) set to 0.05, gave an 88% power to detect a true difference of 0.5 mm.

### Blinding of the operator

The clinical examiner (DFB) was tested towards the smoking status of a sample of 30 chronic periodontitis patients, 16 smokers (>10 cigarettes/day for at least 5 years) and 14 non‐smokers (never or not in the last 5 years). Calculus, plaque, and staining were removed and after rinsing with 0.2% chlorhexidine gluconate (Corsodyl, GlaxoSmithKline, London, UK) for 1 min, the examiner, wearing a face mask, scored the smoking status as yes or no. Twenty‐eight patients (93%) were correctly identified as either non‐smokers or smokers (*p *<* *0.001).

### Study group

Eighty patients, 40 smokers and 40 non‐smokers, with moderate to severe chronic periodontitis (Armitage 1999) referred for periodontal treatment from general practitioners in a rural district of Norway, were enrolled in this single‐arm clinical trial March 2012 through September 2013 (Table 1). A detailed medical, dental, periodontal, and smoking history for the patients was obtained from clinical examinations (including weight and height registrations), health forms, questionnaires, and by consulting their physicians. Furthermore, they were examined for eligibility and consecutively invited to participate.

**Table 1:** Baseline (T0) patient characteristics by smoking status. Frequency/distribution of participants by sociodemographic and clinical characteristics, and smoking status. *n *=* *80

	Smokers, *n *=* *40	Non‐smokers, *n *=* *40	*p‐*level
Age (*n*/%)			
<60 years	19/47.5	18/45	0.727
≥60 years	21/52.5	22/55	
Gender (*n*/%)			
Male	15/37.5	23/57.5	0.121
Female	25/62.5	17/41.5	
Marital status (*n*/%)			
Married or cohabitant	24/60.0	35/87.2	0.011
Single	16/40.0	5/12.8	
Income (*n*/%)			
Yes	18/45.0	28/70.0	0.069
No	22/55.0	12/30.0	
Education (*n*/%)			
≤9 years	30/75.0	20/50.0	0.025
>9 years	10/25.0	20/50.0	
Satisfaction with oral health (*n*/%)			
Content	10/25.6	14/35.9	0.368
Neither discontent or content, or discontent	28/74.4	25/64.1	
BMI (mean ± SD)	24.3 (4.1)	25.7 (2.9)	0.112
Number of teeth present (mean ± SD)	23.4 (5.2)	25.1 (2.9)	0.069
BI (mean ± SD)	66.7 (18.2)	67.3 (15.7)	0.865
PI (mean ± SD)	54.6 (21.9)	57.1 (20.3)	0.610
PD (mean ± SD)	3.8 (1.5)	3.4 (1.4)	0.028
CAL (mean ± SD)	4.6 (1.8)	4.0 (1.5)	<0.001

BMI, body mass index; BI, bleeding index; PI, plaque index; PD, probing depth; CAL, clinical attachment level.

The inclusion criteria were healthy subjects between 35 and 75 years, with no medication that could affect periodontal healing, having at least four non‐adjacent teeth with inter‐proximal PD ≥ 6 mm and clinical attachment loss ≥5 mm, bleeding on probing (BoP), and no signs of apical pathology (Tonetti & Claffey 2005, Page & Eke 2007). The subjects were either smokers (>10 cigarettes/day for at least 5 years) or non‐smokers (never or not in the last 5 years). Exclusion criteria were any current medical condition affecting periodontal treatment and the use of systemic antibiotics or subgingival scaling in the 6 months before initiation of the study.

### Smoking status

The subjectively reported smoking status was calculated in pack years; the number of cigarettes smoked daily multiplied by the number of years divided by 20 (a standard pack of cigarettes) (Scott et al. 2001). Before treatment, smoking status was objectively validated by measuring cotinine levels in serum. Peripheral venous blood was collected from each participant in a glass vacutainer. After coagulation, blood was centrifuged (1000 rpm/10 min) and the serum was stored in aliquots at −80°C. Serum cotinine was assessed according to the instructions of the serum enzyme immunoassay kit (Cotinine ELISA Kit, MyBioSource, San Diego, USA) by measuring the absorbance at 450 nm with a microplate reader (FluoStar Optima V1.32 R2, BMG Labtech, Offenburg, Germany).

### Treatment

**Figure 1:**
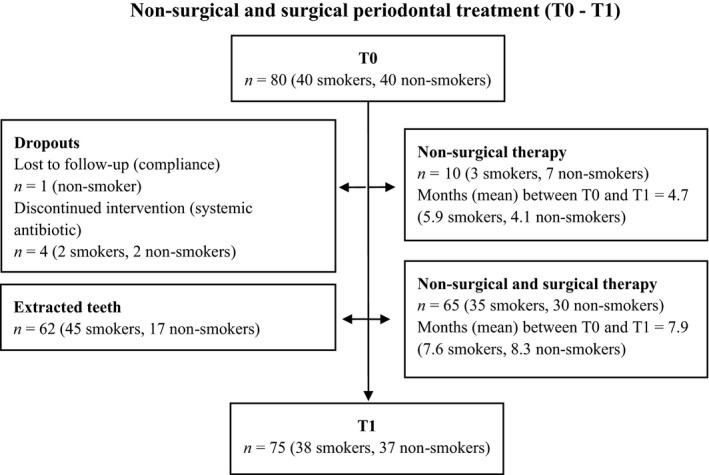
Study flow chart.

Non‐surgical and surgical periodontal treatment was performed by one operator (DFB). All patients were subjected to non‐surgical treatment consisting of motivation and instruction in oral hygiene and debridement using hand instrumentation (Hu‐Friedy, Chicago, IL, USA; and American Eagle Instruments, Missoula, MT, USA) under local anaesthesia. Teeth with hopeless prognosis were extracted during the non‐surgical treatment phase (Mcguire 1991). Each treatment session lasted 60–90 min and mean number of treatment sessions was 5.5 for smokers and 5.0 for non‐smokers. The smokers were motivated for smoking cessation and encouraged to participate in a public smoking cessation program (Røyketelefonen, Helsedirektoratet, Oslo, Norway). Sixteen patients (40%) accepted. After a healing period of 8 weeks, re‐evaluation was performed (Segelnick & Weinberg 2006). To further reduce PD and inflammation in patients presenting PD >5 mm with BoP and exhibiting adequate oral hygiene routines, periodontal surgery was implemented. Sixty‐five patients, 35 smokers and 30 non‐smokers, received periodontal surgery (Fig. 1). Mean number of surgeries per patient was 2.0 for smokers and 1.8 for non‐smokers. Both periodontal flap and gingivectomy techniques were used following standard protocols. Sutures and periodontal dressings were removed at 7–10 days. A 0.2% chlorhexidine gluconate rinse (Corsodyl, GlaxoSmithKline, London, UK) was implemented for 4 weeks postsurgery. Postsurgical controls, including full mouth plaque removal and oral hygiene instruction, were conducted every second or third week until clinical evaluation at 12 weeks.

### Clinical assessment

Before clinical examination, a full mouth series of intra‐oral radiographs was taken. Clinical measurements were registered at baseline pre‐treatment (T0) and at 3 months post‐treatment (T1). PD was recorded as the distance in mm from the gingival margin to the probeable base of the pocket, and CAL as the distance in mm from the cemento‐enamel junction or the margin of a dental restoration to the probeable base of the pocket. PD and CAL were measured with a manual periodontal probe (PCPUNC 15, Hu‐Friedy, Chicago, IL, USA) at six sites per tooth rounding up to the nearest mm. Full mouth gingival bleeding was recorded as the percentage of sites showing bleeding after gentle probing (Ainamo & Bay 1975) and full mouth dental plaque as the percentage of tooth surfaces with visible plaque following staining with disclosing solution (O'Leary et al. 1972). As a supplement to staining, the periodontal probe was used to discriminate between plaque and pellicle.

### Microbiological assessment

At T0, two sterile paper points were inserted into the deepest periodontal pocket in each patient, and the procedure was repeated at the same site at T1. Before sampling, the site was carefully cleaned of supragingival plaque and kept dry. The paper points were gently inserted towards the apex of the pocket and kept in place for 20 sec (Renvert et al. 1992, Belibasakis et al. 2014) before removal and immersion into a pre‐reduced sterile transport medium (PRAS Dental Transport Medium, Morgan Hill, CA, USA). The sample tubes were analysed at Microbiological Diagnostic Service, Department of Oral Biology, Faculty of Dentistry, University of Oslo, Oslo, Norway by DNA‐DNA hybridization (checkerboard technique) (Socransky et al. 2004). The results were reported separately for each sample, showing both qualitative and quantitative results. Analysis included detection of red (*Porphyromonas gingivalis*,* Treponema denticola,* and *Tannerella forsythia*) and orange (*Prevotella intermedia*,* Prevotella nigrescens*,* Fusobacterium nucleatum subsp polymorphum, Fusobacterium nucleatum subsp nucleatum, Fusobacterium nucleatum subsp vincentii, and Parvimonas micra*) complex species, and *Aggregatibacter actinomycetemcomitans* (Socransky et al. 1998).

### Statistical analysis

Normality assumptions of the continuous variables were performed using the skewness and kurtosis test. Descriptive statistics were executed using frequencies and percentage for qualitative variables (chi‐square test) and mean ± standard deviation for quantitative variables (ordinary two sample *t*‐test and Mann–Whitney test).

PD ≥5 mm with BoP, defined as the primary outcome variable, was dichotomized as (1) present and (0) absent. In the logistic regressions each site, corrected for clustering of the data within teeth and patients, was the unit of the analysis. Patient‐related explanatory variables were tested in unadjusted models and in a multiple model adjusted for covariates. In the analysis, time was categorized as T0 (0) and T1 (1), age as ˂60 years (0) and ≥60 years (1), gender as male (0) and female (1), self‐reported education as ≥9 years (0) and <9 years (1), marital status as living alone (0) and married/cohabitant (1), and number of teeth at T0 < 15 teeth (0) and ≥15 teeth (1). For each patient an overall mean value for PD, CAL, BI, and PI was calculated at T0. This measure was applied to adjust for heterogeneity at T0. Sites presenting PD ≥5 mm with BoP at teeth extracted between T0 and T1 were not included in the analysis. Odds ratios (OR) and 95% confidence intervals (95% CI) were calculated.

Secondary outcome variables, changes in plaque index (PI), bleeding index (BI), PD, and amount of bacteria, were analysed by conventional regression analysis, corrected for clustered observations. A *p*‐value of less than 0.05 was considered statistically significant. All analyses were conducted using Stata version 13 (Stata Corp., College Station, TX, USA).

## Results

**Figure 2:**
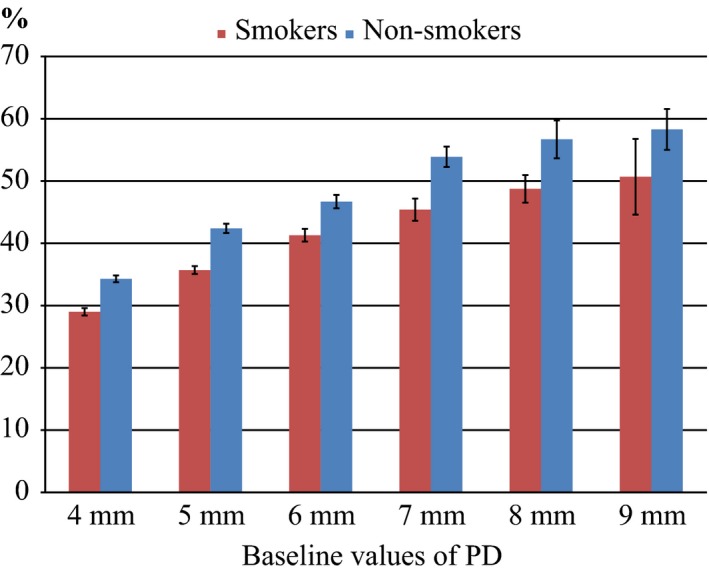
Mean percentage of sites showing probing depth reduction of one mm or more between T0 and T1 for each probing depth category.

Patient characteristics are summarized in Table 1. Eighty patients were included; 40 smokers [mean age 57.6 years (range 37–70)] and 40 non‐smokers [mean age 58.7 years (range 35–73)]. Seventy‐five patients (94%) completed the study. During treatment, significantly more teeth with hopeless prognosis were extracted in the smoking group (*p *=* *0.009); 37 teeth in 16 smokers and 11 in nine non‐smokers. In both groups mean PD, plaque, and bleeding index were significantly reduced (*p *<* *0.001) with no differences between the groups at T0 or T1. In smokers, mean PD was reduced from 3.8 to 2.6 mm (1.2 mm) and in non‐smokers from 3.4 to 2.3 mm (1.1 mm). Figure 2 presents mean percentage of sites showing PD reduction of one mm or more between T0 and T1 for each PD category for smokers and non‐smokers. Compared with non‐smokers, smokers demonstrated between 5 % and 8 % less number of sites with mm reduction for PD categories between four and nine mm.

The distribution of PD ≥5 mm with BoP, at T0 and T1 is summarized in Table 2. At T0, smokers presented with 1471 (26%) and non‐smokers with 1049 (18%) sites with PD ≥5 mm and BoP. The numbers decreased to 132 (3%) sites for smokers and 52 (1%) sites for non‐smokers at T1. At the patient level, the mean number of sites with PD ≥5 mm and BoP per smoker was 36.8 (26%) and 26.3 (18%) per non‐smoker at T0 (not tabulated). The corresponding estimates at T1 were 3.47 (3%) and 1.41 (1%). At T1 no patients in the non‐smoking group presented with more than seven sites with PD ≥5 mm with BoP, whereas five smokers exhibited 10 or more sites. These five patients were all heavy smokers with a mean cotinine level of 725 ng/ml (range 501–861). Mean cigarette consumption in the smoking group was 37 pack years (20–108) and mean cotinine level 471 ng/ml (range 168–861). No patients reported starting or quitting smoking during the study.

**Table 2:** Numbers of PD ≥ 5 mm with BoP by smoking status before (T0) and following periodontal treatment (T1) at arch and tooth level

Localization within arch, tooth and site	T0			T1		
	Smokers n (%)	Non‐smokers n (%)	*p*	Smokers n (%)	Non‐smokers n (%)	*p*
Overall	1471 (26.4)	1049 (17.5)	<0.001	132 (2.6)	52 (1.0)	<0.001
Maxilla	894 (32.9)	638 (20.9)	<0.001	78 (3.2)	32 (1.2)	<0.001
Buccal	345 (26.4)	260 (17.1)	0.001	26 (2.1)	9 (0.7)	0.002
Palatal	549 (40.4)	378 (24.8)	<0.001	52 (4.3)	23 (1.7)	0.001
Multi‐rooted	306 (46.4)	334 (40.1)	0.159	37 (7.1)	22 (3.1)	0.017
Buccal	131 (39.7)	136 (32.6)	0.105	10 (3.8)	7 (2.0)	0.209
Palatal	175 (53.0)	198 (47.5)	0.309	27 (10.2)	15 (4.2)	0.009
Single‐rooted	588 (28.5)	304 (13.7)	0.110	41 (2.1)	10 (0.8)	0.045
Buccal	214 (20.8)	124 (11.2)	0.680	16 (1.7)	2 (0.2)	0.121
Palatal	374 (46.4)	180 (16.2)	0.030	25 (2.6)	8 (0.8)	0.159
Mandibula	577 (20.3)	411 (13.9)	0.009	54 (2.1)	20 (0.7)	0.013
Buccal	253 (17.8)	181 (12.3)	0.027	23 (1.7)	9 (0.7)	0.029
Palatal	324 (22.7)	230 (15.6)	0.009	31 (2.4)	11 (0.8)	0.028
Multi‐rooted	236 (35.8)	211 (30.6)	0.236	21 (3.5)	16 (2.6)	0.538
Buccal	99 (30.0)	86 (24.9)	0.269	9 (3.0)	7 (2.3)	0.617
Lingual	137 (41.5)	125 (36.2)	0.326	12 (4.0)	9 (2.9)	0.561
Single‐rooted	341 (15.6)	200 (8.9)	0.013	33 (1.6)	4 (0.4)	0.191
Buccal	154 (14.1)	95 (8.4)	0.148	14 (1.4)	2 (0.2)	0.628
Lingual	187 (17.1)	105 (9.3)	0.011	19 (1.9)	2 (0.2)	0.190

BoP; bleeding on probing; multi‐rooted, molars; single‐rooted, premolars and incisors; buccal, two proximal‐buccal and one mid‐buccal; palatal, two proximal‐palatal and one mid‐palatal; lingual, two proximal‐lingual and one mid‐lingual.

Between T0 and T1 a higher number of sites with PD ≥5 mm with BoP was removed by tooth extraction in the smoking compared with the non‐smoking group (*p *=* *0.002); 177 and 46 sites, respectively. These sites were excluded from the analysis. Compared with non‐smokers, an overall significantly higher risk was found in smokers to present with PD ≥5 mm with BoP at T1 [OR = 2.01, CI: 1.24, 3.23, *p *=* *0.004 (not tabulated)]. The adjusted analysis withstands the significant association with smoking and PD ≥5 mm with BoP at T1 [OR = 1.90, CI: 1.14, 3.15, *p *=* *0.013 (not tabulated)]. Results of unadjusted and adjusted logistic regression analysis of PD ≥5 mm with BoP are presented in Table 3. For both smokers and non‐smokers the unadjusted analysis showed significant associations between PD ≥5 mm with BoP and mean T0 values of PD, CAL, and BI. For smokers, a significant association was shown for mean number of teeth at T0. For both groups the adjusted analysis revealed significant associations between PD ≥5 mm with BoP and mean baseline values of PD, BI, and number of teeth. For smokers only, CAL at T0 and not living alone showed significant associations with the primary outcome.

**Table 3:** Unadjusted and adjusted OR for PD ≥ 5 mm with BoP, stratified by smoking status

Characteristics	Smokers						Non‐smokers					
	Unadjusted^a^			Adjusted^b^			Unadjusted^a^			Adjusted^b^		
	OR	95% CI	*p*	OR	95% CI	*p*	OR	95% CI	*p*	OR	95% CI	*p*
Time												
T0	1			1			1			1		
T1	0.17	0.13, 0.22	<0.001	0.086	0.06, 0.12	<0.001	0.085	0.06, 0.13	<0.001	0.041	0.03, 0.06	<0.001
Age												
<60 years	1			1			1			1		
≥60 years	0.93	0.62, 1.39	0.710	1.00	0.99, 1.01	0.350	0.75	0.48, 1.16	0.196	0.98	0.98, 0.99	<0.001
Gender												
Male	1			1			1			1		
Female	0.97	0.63, 1.50	0.904	0.88	0.74, 1.05	0.154	1.33	0.90, 1.98	0.152	1.03	0.84, 1.27	0.754
Education												
≥9 years	1			1			1			1		
<9 years	1.30	0.86, 2.00	0.208	1.07	0.89, 1.29	0.488	1.35	0.94, 1.95	0.106	0.87	0.71, 1.05	0.150
Marital status												
Single	1			1			1			1		
Co‐habitant	1.04	0.70, 1.55	0.855	0.84	0.71, 0.98	0.027	1.40	0.88, 2.22	0.151	0.90	0.63, 1.28	0.545
Teeth at T0												
˂15 teeth	1											
≥15 teeth	0.95	0.92, 0.99	0.015	1.07	1.03, 1.10	<0.001	0.99	0.94, 1.05	0.806	0.96	0.94, 0.98	<0.001
PD, per mm increase	3.10	2.70, 3.56	<0.001	3.54	2.95, 4.24	<0.001	3.42	2.98, 3.92	<0.001	4.36	3.74, 5.09	<0.001
CAL, per mm increase	1.78	1.60, 1.99	<0.001	1.14	1.04, 1.26	0.007	2.17	1.93, 2.42	<0.001	1.02	0.87, 1.20	0.761
BI, per 10% increase	1.15	1.03, 1.27	0.009	1.09	1.05, 1.13	<0.001	1.29	1.16, 1.44	<0.001	1.14	1.07, 1.22	<0.001
PI, per 10% increase	1.07	0.96, 1.19	0.225	1.05	0.99, 1.10	0.083	1.10	1.00, 1.21	0.062	0.97	0.94, 1.01	0.115

BoP, bleeding on probing; CI, Confidence Interval; OR, Odds Ratio; PD, probing depth; CAL, clinical attachment level; BI, bleeding index; PI, plaque index.

a Unadjusted logistic regression for PD ≥ 5 mm with BoP.

b Adjusted logistic regression for PD ≥ 5 mm with BoP, and time, age, gender, education, marital status, teeth at T0, and mean values of PD, CAL, BI, and PI at T0. Sites with PD ≥ 5 mm with BoP extracted between T0 and T1 were not included in the analysis.

Plaque‐positive and plaque‐negative sites were analysed for the association of PD ≥5 mm with BoP in the adjusted model, with plaque‐negative sites in non‐smokers as reference category. Plaque‐positive sites in smokers had an overall higher risk to present with PD ≥5 mm with BoP at T1 compared with plaque‐positive sites in non‐smokers [4.14, CI:2.16, 7.96, *p *< 0.001 and OR:3.09, CI:1.65, 5.79, *p* ˂ 0.001, respectively (not tabulated)]. Further, within arch, teeth, and site, a highly significant effect of plaque was found with an interaction between plaque and smoking (Table 4). Presence of plaque more than doubled the risk of having PD ≥5 mm with BoP in smokers compared with non‐smokers (OR= 4.98, CI: 2.50, 9.93, *p *<* *0.001 and OR = 2.40, CI: 1.09, 5.30, *p *=* *0.030, respectively) at maxillary molar palatal sites.

**Table 4:** Relative risks for PD ≥ 5 mm with BoP at arch, tooth, and site levels, with and without cigarette smoking and presence of plaque following periodontal treatment. Reference category: plaque‐negative sites in non‐smokers

Localization	Smokers plaque‐negative sites			Non‐smokers plaque‐positive sites			Smokers plaque‐positive sites		
	OR	CI	*p*	OR	CI	*p*	OR	CI	*p*
Maxilla	1.10	0.94, 1.29	0.224	2.21	1.22, 4.00	0.009	3.16	1.65, 5.90	<0.001
Mandibula	1.03	0.82, 1.30	0.783	7.12	2.47, 20, 52	<0.001	8.75	3.02, 25.33	<0.001
Multi‐rooted	1.15	0.98, 1.37	0.097	2.92	1.51, 5.67	0.002	4.13	2.17, 7.87	<0.001
Single‐rooted	1.01	0.81, 1.26	0.946	2.59	0.91, 7.38	0.075	3.96	1.51, 10.33	0.005
Buccal	1.04	0.85, 1.26	0.730	2.09	1.05, 4.18	0.037	3.07	1.47, 6.40	0.003
Palatal/lingual	1.08	0.91, 1.29	0.371	4.27	2.13, 8.56	<0.001	5.24	2.67, 10.30	<0.001
Teeth within arch									
Maxilla									
Multi‐rooted	1.25	1.02, 1.53	0.029	1.64	0.84, 3.19	0.148	3.15	1.53, 6.49	0.002
Single‐rooted	1.01	0.79, 1.29	0.921	2.87	0.99, 1.29	0.053	3.27	1.25, 8.51	<0.001
Mandibula									
Multi‐rooted	1.05	0.83, 1.32	0.712	12.64	4.12, 38.26	<0.001	10.87	4.18, 28.26	<0.001
Single‐rooted	1.00	0.71, 1.39	0.996	2.04	0.44, 9.41	0.361	5.50	1.37, 22.00	0.016
Sites within arch									
Maxilla									
Buccal	0.99	0.80, 1.22	0.920	1.17	0.58, 2.36	0.665	2.00	0.89, 4.49	0.095
Palatal	1.21	0.99, 1.47	0.066	3.59	1.80, 7.14	<0.001	4.45	2.38, 8.33	<0.001
Mandibula									
Buccal	1.15	0.82, 1.59	0.420	6.46	2.26, 18.53	0.001	7.79	2.54, 23.85	<0.001
Lingual	0.97	0.71, 1.33	0.844	7.66	2.19, 26.83	0.001	9.37	2.84, 30.97	<0.001
Sites within multi‐rooted teeth									
Maxilla									
Buccal	1.31	1.01, 1.72	0.045	0.98	0.45, 2.13	0.964	1.54	0.50, 4.79	0.456
Maxilla									
Palatal	1.21	0.92, 1.58	0.180	2.40	1.09, 5.30	0.030	4.98	2.50, 9.93	<0.001
Mandibula									
Buccal	1.16	0.76, 1.77	0.503	11.41	3.04, 42.77	<0.001	9.88	3.01, 32.39	<0.001
Mandibula									
Lingual	0.96	0.70, 1.33	0.823	15.10	4.60, 49.61	<0.001	12.69	4.27, 37.73	<0.001
Sites within single‐rooted teeth									
Maxilla									
Buccal	0.85	0.61, 1.19	0.337	1.17	0.26, 5.17	0.840	2.85	0.87, 9.32	0.083
Maxilla									
Palatal	1.13	0.86, 1.48	0.390	5.05	1.47, 17.34	0.010	3.56	1.31, 9.67	0.013
Mandibula									
Buccal	1.08	0.72, 1.61	0.709	2.44	0.48, 12.54	0.284	5.26	1.10, 25.23	0.038
Mandibula									
Lingual	0.96	0.61, 1.51	0.871	1.78	0.31, 10.42	0.521	5.17	1.23, 21.58	0.024

BoP, bleeding on probing; CI, confidence interval; OR, odds ratio; multi‐rooted, molars; single‐rooted, premolars and incisors; palatal, mesio‐palatal and palatal and disto‐palatal; lingual, mesio‐ling and lingual and disto‐lingual; buccal, mesio‐buccal and buccal and disto‐buccal.

Logistic regression with outcome PD ≥ 5 mm with BoP adjusted for time, age, gender, education, marital status, mean PD, CAL, BI, BI, and number of teeth present at T0. Sites with PD ≥ 5 mm with BoP extracted between T0 and T1 were not included in the analysis.

**Figure 3:**
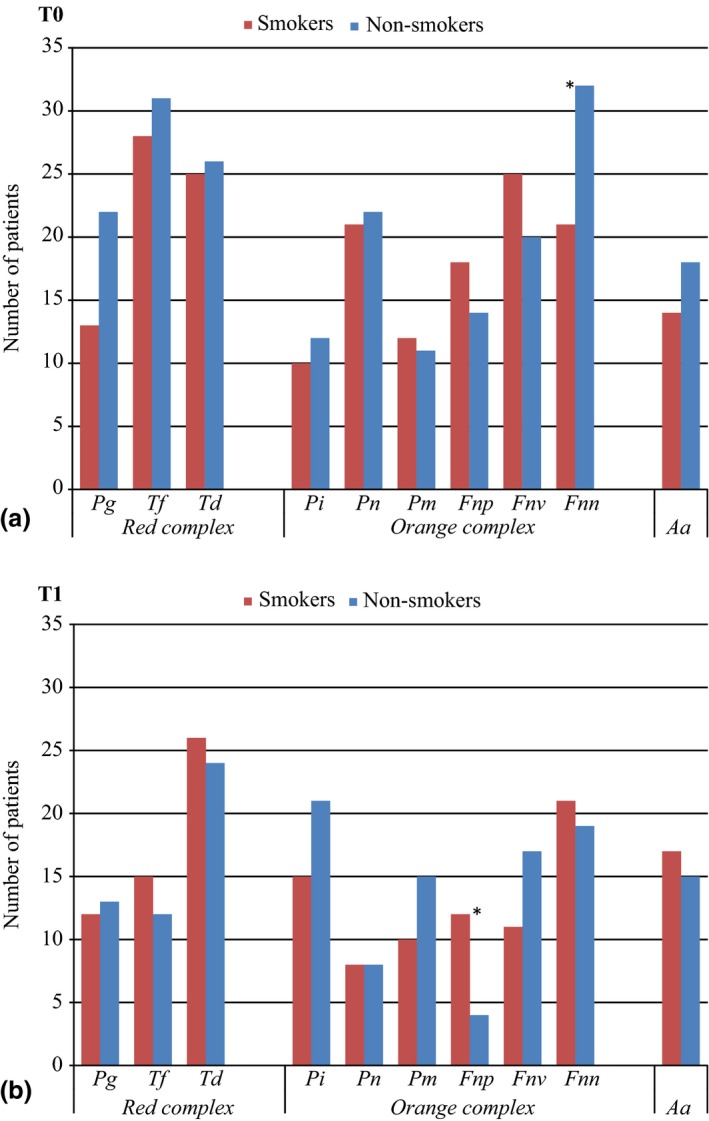
(a) Number of patients harbouring different pathogens at baseline. (b) Number of patients harbouring different pathogens following non‐surgical and surgical periodontal therapy. *Statistical significant differences between the groups.

Figure 3 shows number of patients harbouring target microbial species at T0 and T1. Mean sample site PDs did not differ between smokers and non‐smokers at T0 (7.4 mm) and T1 (3.7 mm). No significant quantitative reduction was observed for the red (*p *=* *0.35) and orange (*p *=* *0.16) complex species in the smoking group (not tabulated). Nevertheless, a significant reduction of *T. forsythia* (*p *=* *0.038), *P. nigrescens* (*p *=* *0.035), and *F. nucleatum subsp vincentii* (*p *<* *0.001) was detected. Among non‐smokers a significant reduction was observed for the red complex species (*p *=* *0.010), specifically for *P. gingivalis* (*p* = 0.013) and *T. forsythia* (*p *=* *0.005). The orange complex species showed a borderline significant reduction (*p *=* *0.060) with a significant reduction of *P. intermedia* (*p *=* *0.008), *P. nigrescens* (*p *=* *0.004), and *F. nucleatum subsp polymorphum* (*p* = 0.035). However, differences detected comparing single species were considered inconclusive due to multiple testing.

## Discussion

This prospective study appears to be the first to compare the effect of non‐surgical and surgical periodontal therapy in smokers and non‐smokers using clinical and microbiological parameters and an objective validation of self‐reported smoking habits. In both the smoking and non‐smoking groups, PD, BI, and PI improved significantly following treatment. PD categories between 4 and 8 mm showed a less reduction in smokers compared with non‐smokers. In perspective, Tomasi reported a 30% reduction in pocket closure in smokers following non‐surgical periodontal therapy with a more limited effect in initially deep pockets (Tomasi et al. 2007). The present study using the same PD categories confirms a less reduction in initially deep pockets in smokers following non‐surgical and surgical treatment. The relatively high number of patients receiving periodontal surgery was due to initially deep PDs in patients exhibiting adequate oral hygiene standards and good general health. Cigarette smoking is not a contraindication to periodontal surgery, but high cigarette consumption is considered a risk factor generating less favourable clinical outcomes (Matuliene et al. 2008). The negative effects of cigarette smoking increase in a non‐linear consumption model and patients consuming ≥ 20 cigarettes per day are considered at high risk for treatment relapse (Lang & Tonetti 2003).

In the present study, the five patients presenting with 10 or more sites with PD ≥5 mm with BoP following treatment were all heavy smokers with a mean serum cotinine level of 725 ng/ml. The discrepancy between measured cotinine level and subjectively reported cigarette consumption was more pronounced for heavy smokers, indicating a higher underreporting. A socioeconomic stigma of smokers and pressure towards smoking cessation, likely make smokers susceptible to underreporting their smoking habits (Scott et al. 2001, Stuber & Galea 2009). This should be a concern for clinicians in the everyday treatment planning and in projecting prognosis for teeth at risk in smokers.

The causality of cigarette smoking on the outcomes of periodontal treatment must be interpreted with caution as smokers tend to present with more advanced periodontitis (Hugoson & Rolandsson 2011). In this study, an association between smoking and PD ≥5 mm with BoP was shown both with and without baseline adjustments. This observation is consistent with a systematic review on the influence of cigarette smoking on the effect of non‐surgical therapy (Labriola et al. 2005). The negative effect of cigarette smoking was shown by including BoP in the primary outcome, although smokers tend to have scarcer bleeding from deeper PDs than non‐smokers (Preber & Bergström 1985). Excluding BoP might positively influence the primary outcome in non‐smokers by deeper probe penetration into the inflamed soft tissue at T0 and a more pronounced shrinkage of gingiva during resolution of the inflammation (Biddle et al. 2001). Further, BoP is considered an indicator for disease progression in high‐risk patients at site level and absence of BoP indicates a lower risk for disease progression in both smokers and non‐smokers (Lang et al. 1990, Claffey & Egelberg 1995).

A significant association was detected between PD ≥5 mm with BoP and presence of plaque, in smokers as well as non‐smokers. This association was highly significant for smokers and particularly pronounced for maxillary molar palatal sites. These sites are immediately exposed to cigarette smoke and thereby to nicotine and combustion products. Binding of nicotine and tar to root surfaces and a 300 times higher concentration of cotinine in GCF compared with plasma, are proposed causative factors for impaired treatment response (Cuff et al. 1989, Ryder et al. 1998, Wan et al. 2009). Further, plaque control being generally demanding in the posterior dentition may increase the probability of cigarette smoke aggravating a plaque‐induced inflammatory process.

An increased presence of red and orange complex species was found in smokers compared with non‐smokers at T0 and T1. The non‐significant reduced counts in the red and the orange complex species, especially *P. gingivalis* and *T. denticola* in smokers, are in agreement with previous reports (Grossi et al. 1997, Haffajee et al. 1997). In the present study, all smokers maintaining elevated red complex bacterial counts at T1 were heavy smokers. In perspective, early dysbiosis in subgingival plaque colonization is influenced by cigarette smoke and in a dose responding manner (Hutcherson et al. 2015). *P. gingivalis* has a potential to enhance early plaque formation in smokers (Bagaitkar et al. 2011, Zeller et al. 2014). Early microbial colonization and poor correlation between the marginal and subgingival ecosystems in smokers might further impair resolution of inflammation during treatment (Joshi et al. 2014). As plaque formation adapts to cigarette smoke and the alterations are reversed when removing the cigarette stimulus, it is critical to avoid smoking exposure during periodontal therapy. The ability of the microbiota to adapt to tobacco exposure should encourage further multilevel investigation of clinical and microbiological effects of smoking cessation or reduction during periodontal treatment.

We acknowledge that the lack of blinding might be a limiting factor in the present study. An attempt to blind the operator with regard to smoking status was unsuccessful. To reduce bias, all data plotting was performed by a person unaware of clinical registrations and smoking status. Moreover, personnel conducting the microbiological analysis were blinded to the smoking status of the patients. To reduce the risk of treatment variation, all patients were treated by one operator. The high‐level oral hygiene standards achieved might be influenced by the Hawthorne effect, as participants were aware of being part of the study.

In conclusion, within limitations of the study, smokers show less favourable treatment response to non‐surgical and surgical periodontal therapy in terms of residual PD ≥5 mm with BoP, reduced counts of red and orange complex bacterial species, and PD reduction. Elevated smoking exposure negatively influenced the number of PD ≥5 mm with BoP and the microbial counts. Correcting for clustered observations within patients and teeth revealed an increased risk for PD ≥5 mm with BoP at plaque‐positive sites in smokers. Collectively, the results demonstrate a site‐specific tissue response in smokers following initial periodontal therapy superimposed on a systemic effect.

**Clinical Relevance**

*Scientific rationale for the study*: Generally, smokers respond less favourable compared with non‐smokers to non‐surgical and surgical periodontal therapy. To predict the outcome of periodontal therapy in smokers, the effect of smoking needs to be critically evaluated at patient, tooth, and site level.

*Principal findings*: Smokers showed impaired clinical and microbiological responses to non‐surgical and surgical periodontal therapy. At tooth and site level, odds ratios for having probing depth ≥5 mm with bleeding on probing following treatment were higher for smokers in all locations, especially for plaque‐positive sites.

*Practical implications*: Clinicians should consider including smoking cessation as a vital component of treatment to optimize the effect of non‐surgical and surgical periodontal therapy.
